# Supplementary material for: Increased expression of a subset of genes within reduced copy number regions across multiple cancer types
Source: bioRxiv. 2026 Apr 14:2026.04.10.717791. Preprint. [Version 1] doi: 10.64898/2026.04.10.717791 (PMC13104823; doi:10.64898/2026.04.10.717791)
Supplement: Supplement 1 [file NIHPP2026.04.10.717791v1-supplement-1.pdf]

bioRxiv preprint doi: https://doi.org/10.64898/2026.04.10.717791; this version posted April 14, 2026. The copyright holder for this preprint (which was not certified by peer review) is the author/funder, who has granted bioRxiv a license to display the preprint in perpetuity. It is made available under aCC-BY 4.0 International license.

Figure S1. TGIF1 copy number reductions in PanCancer datasets.

A) Copy number reductions for TGIF1 are plotted across all TCGA PanCancer datasets (data from cBioPortal). B) Comparison of CNA (loss and gain) for TGIF1 and TGIF2 in the TCGA

bioRxiv preprint doi: https://doi.org/10.64898/2026.04.10.717791; this version posted April 14, 2026. The copyright holder for this preprint (which was not certified by peer review) is the author/funder, who has granted bioRxiv a license to display the preprint in perpetuity. It is made available under aCC-BY 4.0 International license.

PanCancer CRC dataset and in two additional COAD datasets from cBioPortal. q-values for co-occurrence of TGIF1 CNA reduction and TGIF2 CNA gain are shown below.

Figure S2. Analysis of recurrent copy number in TGCA PanCancer datasets.
Average copy number per gene is plotted genome wide for ten TCGA PanCancer datasets. Red lines indicate the average CNA -0.4 cut-off used to select regions, which are indicated in blue. Chromosomes are ordered 1 to 23 (left to right) and are indicated by the orange lines below each plot.

Figure S3. Genome-wide analysis of regions with recurrent copy number loss.
A) For all chromosomes across ten cancer datasets, the number of genes within recurrently deleted regions is shown. The percentage of genes on the chromosome present in the deleted region is shown in parentheses. Only genes that are expressed above an average log2 cut-off of 2 in either normal or tumor in the dataset are included. B) For each of the recurrent copy number loss regions shown in A, the number of genes with higher expression in tumor than normal (T-N log2FC > 1, p-adj < 0.0001) is shown. The number in parentheses indicates the percentage of expressed genes within the region that meet this differential expression cut-off.

Figure S4. Analysis of pairwise overlap between upregulated genes among cancer types.
For all pairs of cancers with a shared copy number reduced region where there were at least 15 genes with higher expression in tumor than normal (at the more relaxed cutoff: T-N log2FC > 0.4, p-adj < 0.01), the significance of the overlap was analyzed (hypergeometric test). A and B show all pairwise comparisons for the regions on chromosome 8 (A) and chromosome 17 (B). Panel C shows a similar analysis for all other regions with at least 15 genes upregulated in each of the indicated pairs. Cancer pairs are shown on the left (the chromosomal region is also shown for C). Data is plotted as fold difference comparing the observed overlap to the expected, with the size of each dot representing the p-value. The size key for all p-values is shown in A: p < 0.01 shown in red, p >= 0.01 in gray.

Figure S5. Genes with higher expression in tumor than normal are scattered across deletion regions.
Average copy number for all expressed genes within recurrent copy number loss regions on chromosome 10 (GBM and LUSC), chromosome 16 (BRCA) and chromosome 19 (LUAD) is plotted. The positions of genes with higher expression in tumor than normal (T-N log2FC > 1, p-

bioRxiv preprint doi: https://doi.org/10.64898/2026.04.10.717791; this version posted April 14, 2026. The copyright holder for this preprint (which was not certified by peer review) is the author/funder, who has granted bioRxiv a license to display the preprint in perpetuity. It is made available under aCC-BY 4.0 International license.

adj < 0.0001) is shown in red. The number of genes meeting the differential expression cut-off is shown for each plot. Note that different amounts of chromosome 10 are plotted for GBM and LUSC, since the recurrent copy number reduction is different in these two datasets, encompassing 100% of the chromosome in GBM but only ~46% in LUSC. These four regions were selected since they were relatively large and included higher numbers of genes with higher expression in the tumor samples.

Figure S6. Gene expression changes and copy number variation.
Each dataset was divided by copy number (loss: < -0.4, gain: > +0.4, NC: -0.4 to +0.4) and average T-N log2 gene expression difference was plotted for each of the indicated gene sets: A) Glycolysis, B) Hypoxia, C) EMT. p-values are for the comparison of average log2FC between the gain and loss categories. Plotted in red is the average for each bin and the trendline based on the average.

bioRxiv preprint doi: https://doi.org/10.64898/2026.04.10.717791; this version posted April 14, 2026. The copyright holder for this preprint (which was not certified by peer review) is the author/funder, who has granted bioRxiv a license to display the preprint in perpetuity. It is made available under aCC-BY 4.0 International license.

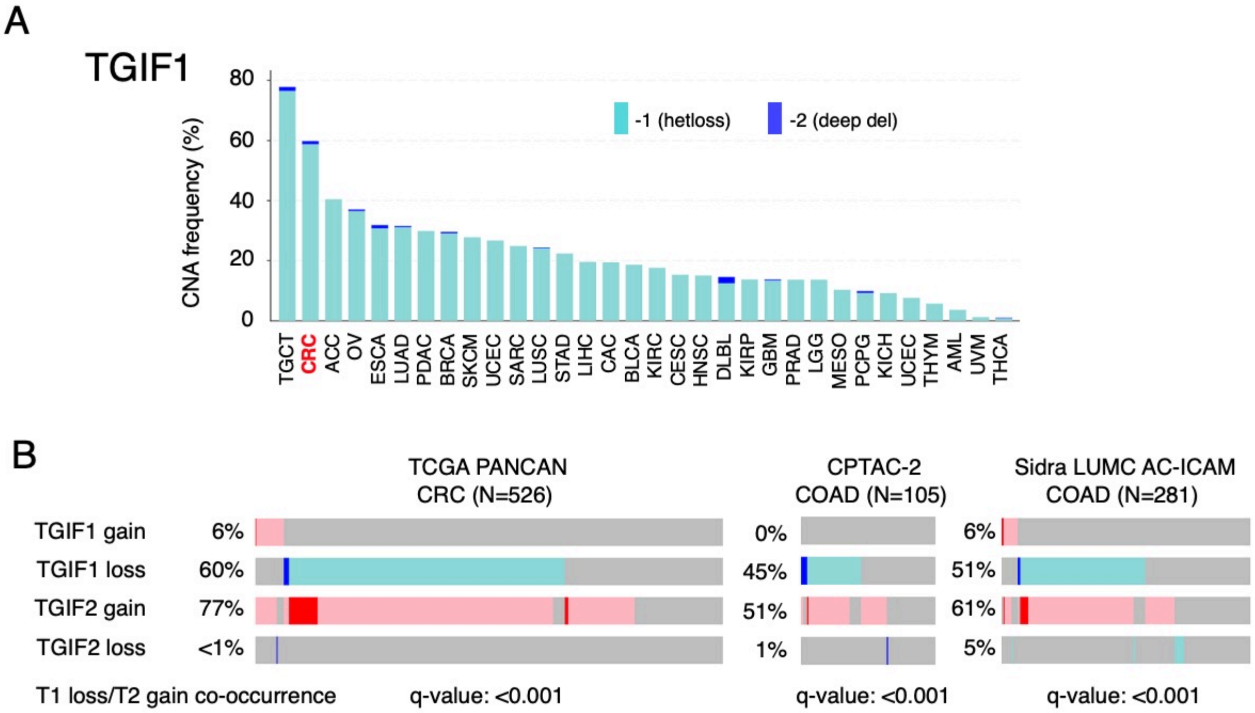

Supplementary Figure 1

bioRxiv preprint doi: https://doi.org/10.64898/2026.04.10.717791; this version posted April 14, 2026. The copyright holder for this preprint (which was not certified by peer review) is the author/funder, who has granted bioRxiv a license to display the preprint in perpetuity. It is made available under aCC-BY 4.0 International license.

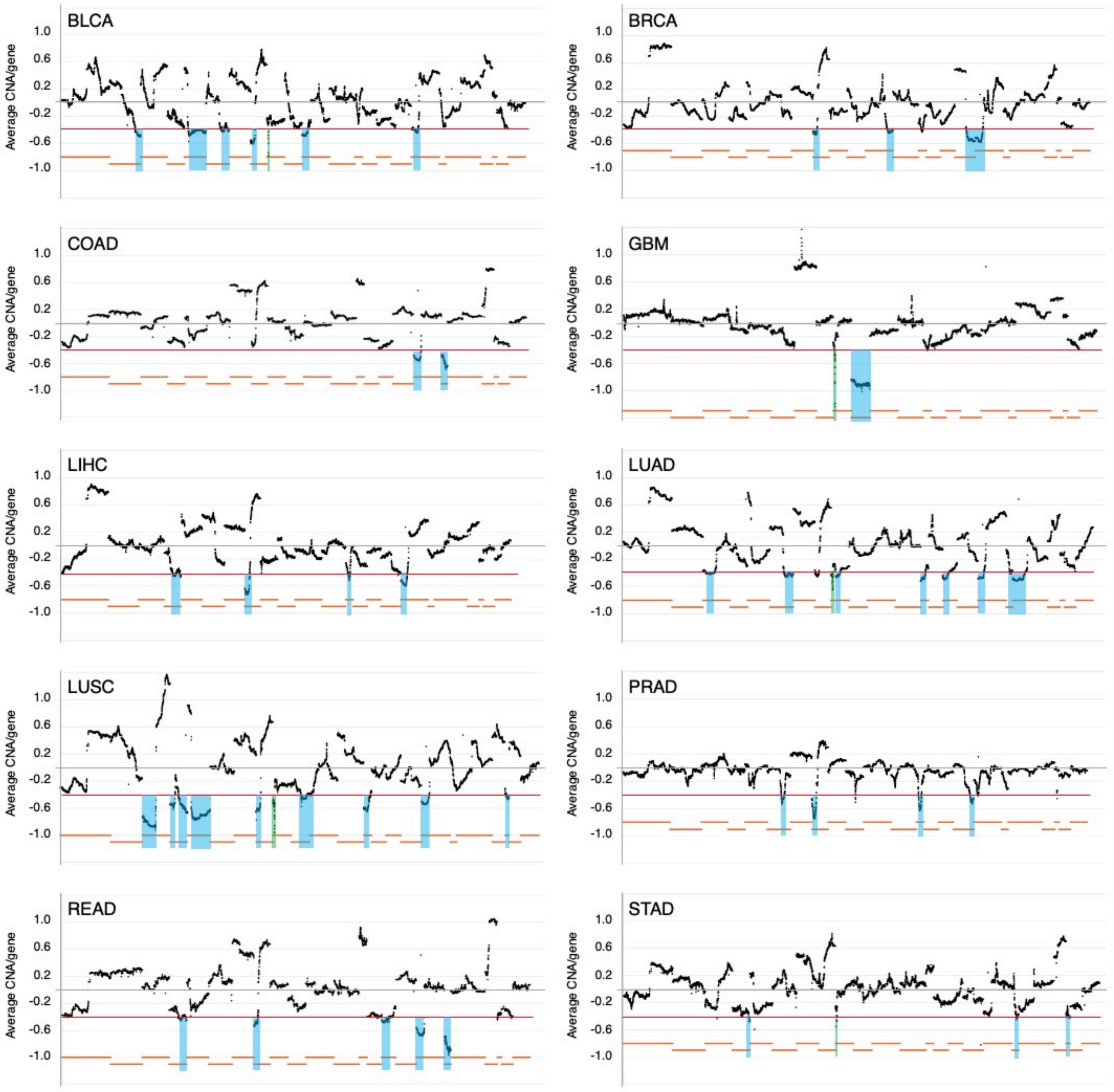

Supplementary Figure 2

bioRxiv preprint doi: https://doi.org/10.64898/2026.04.10.717791; this version posted April 14, 2026. The copyright holder for this preprint (which was not certified by peer review) is the author/funder, who has granted bioRxiv a license to display the preprint in perpetuity. It is made available under aCC-BY 4.0 International license.

A

Chr	BLCA	BRCA	COAD	GBM	LIHC	LUAD	LUSC	PRAD	READ	STAD
1										
2	172 (18)									
3						222 (26)	423 (49)			
4					261 (50)		443 (81)		134 (25)	38 (7)
5	551 (81)						586 (84)			
6	87 (12)					284 (39)		93 (13)		
7										
8	133 (27)	137 (27)			167 (35)	139 (27)	143 (28)	141 (28)	139 (28)	
9						142 (25)				
10				577 (100)			264 (46)			
11	180 (21)	175 (20)					62 (7)			
12										
13					72 (32)	152 (62)	213 (87)	92 (38)		
14										
15						190 (42)			249 (56)	
16		280 (44)						119 (19)		
17	223 (26)	247 (28)	243 (28)		207 (25)	201 (23)	252 (28)		248 (28)	
18			195 (100)			96 (49)			197 (100)	97 (48)
19						385 (35)				
20										
21							114 (85)			67 (50)
22										
X										

B

Chr	BLCA	BRCA	COAD	GBM	LIHC	LUAD	LUSC	PRAD	READ	STAD
1										
2	8 (4.65)									
3						14 (6.31)	24 (5.67)			
4					16 (6.13)		34 (7.67)		4 (2.99)	2 (5.26)
5	19 (3.45)						54 (9.22)			
6	2 (2.30)					15 (5.28)		0 (0.00)		
7										
8	5 (3.76)	8 (5.84)			12 (7.19)	11 (7.91)	10 (6.76)	4 (2.84)	4 (2.88)	
9						6 (4.23)				
10				26 (4.51)			33 (13.41)			
11	7 (3.89)	5 (2.86)					8 (12.90)			
12										
13					4 (5.56)	8 (5.26)	26 (12.21)	2 (2.17)		
14										
15						25 (13.16)			6 (2.41)	
16		19 (6.79)						6 (5.04)		
17	11 (4.93)	7 (2.83)	10 (6.99)		5 (2.42)	13 (6.47)	22 (8.73)		2 (0.81)	
18			11 (5.64)			8 (8.33)			3 (1.52)	6 (6.19)
19						25 (6.49)				
20										
21							16 (14.04)			3 (4.48)
22										
X										

Supplementary Figure 3

bioRxiv preprint doi: https://doi.org/10.64898/2026.04.10.717791; this version posted April 14, 2026. The copyright holder for this preprint (which was not certified by peer review) is the author/funder, who has granted bioRxiv a license to display the preprint in perpetuity. It is made available under aCC-BY 4.0 International license.

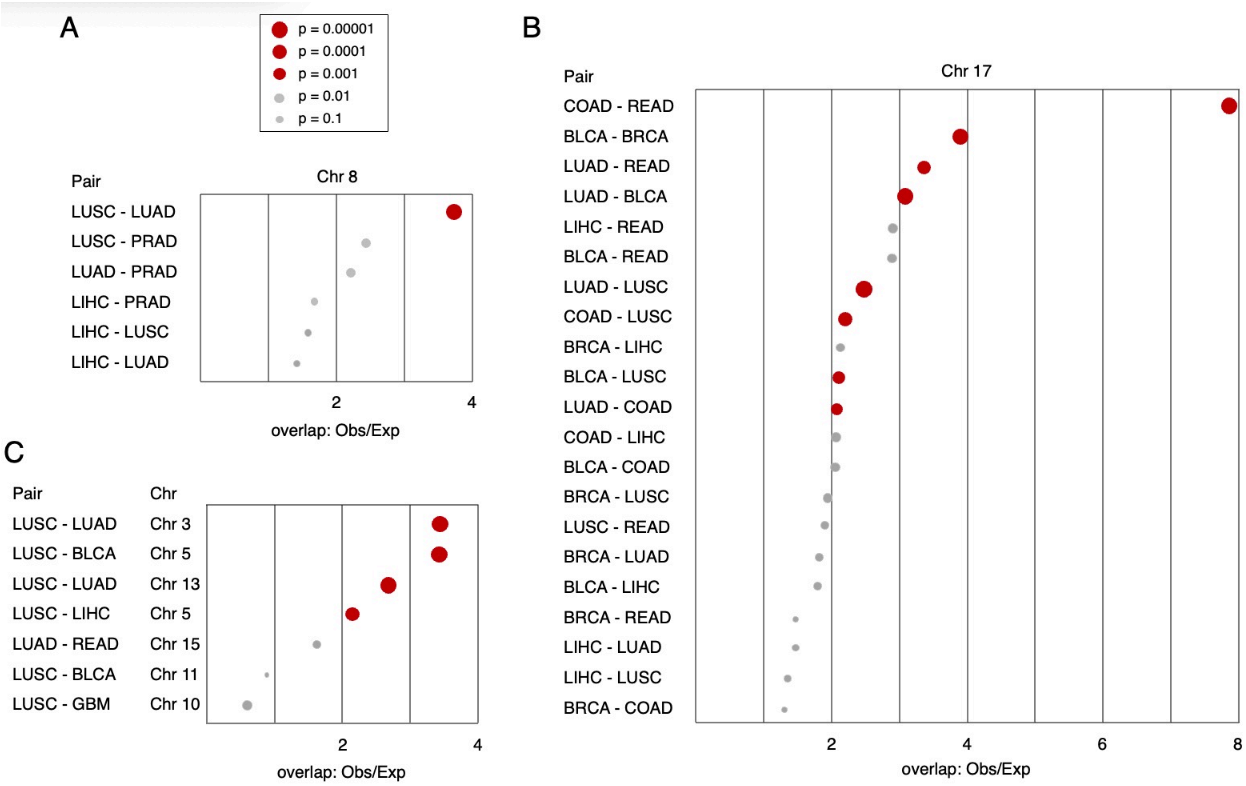

Supplementary Figure 4

bioRxiv preprint doi: https://doi.org/10.64898/2026.04.10.717791; this version posted April 14, 2026. The copyright holder for this preprint (which was not certified by peer review) is the author/funder, who has granted bioRxiv a license to display the preprint in perpetuity. It is made available under aCC-BY 4.0 International license.

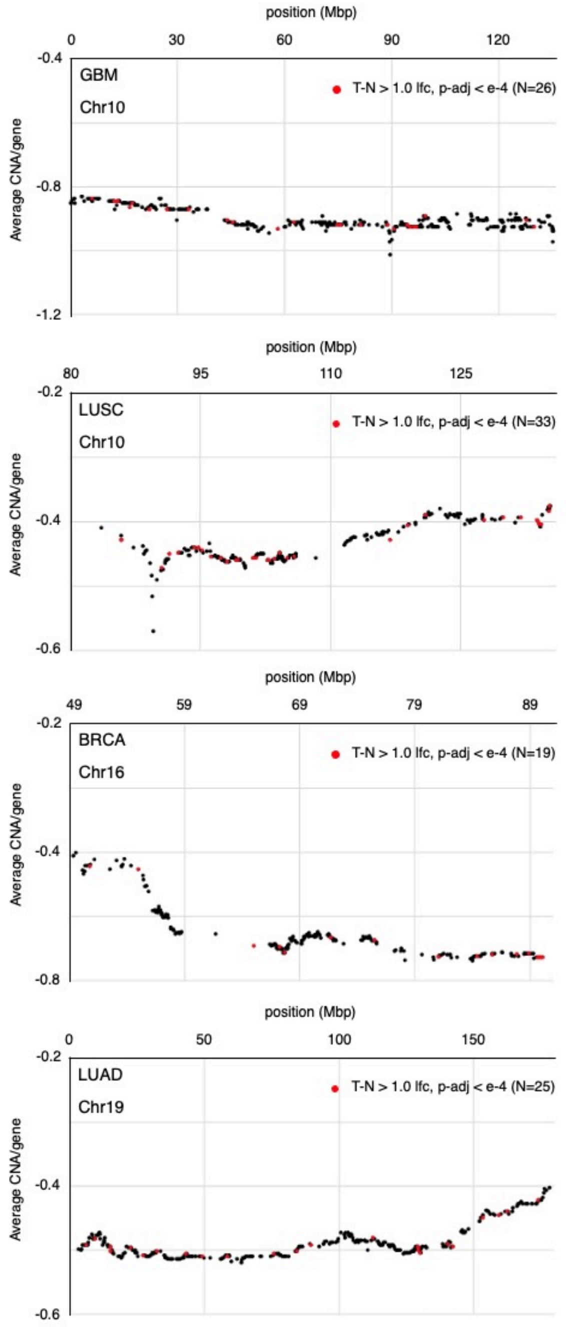

Supplementary Figure 5

bioRxiv preprint doi: https://doi.org/10.64898/2026.04.10.717791; this version posted April 14, 2026. The copyright holder for this preprint (which was not certified by peer review) is the author/funder, who has granted bioRxiv a license to display the preprint in perpetuity. It is made available under aCC-BY 4.0 International license.

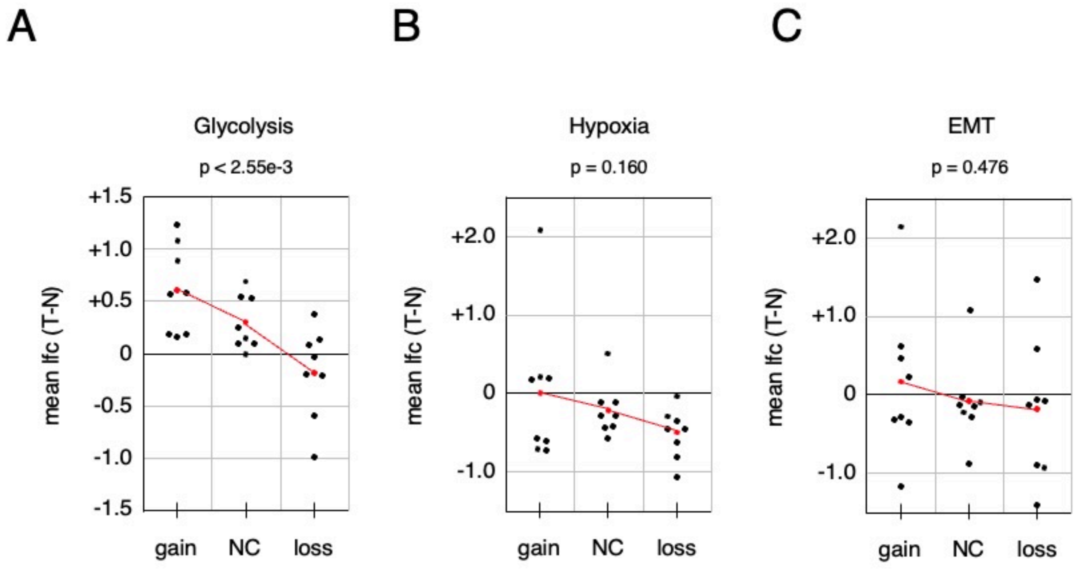

Supplementary Figure 6
